# MRI in the diagnosis and management of epileptomas

**DOI:** 10.1111/epi.12442

**Published:** 2013-12-12

**Authors:** John S Duncan, Jane de Tisi

**Affiliations:** Department of Clinical and Experimental Epilepsy, UCL Institute of NeurologyLondon, United Kingdom

**Keywords:** MRI, Functional MRI, Glioma, Epilepsy surgery, Tractography

## Abstract

High-resolution magnetic resonance imaging (MRI) is invaluable for identifying cerebral tumors that cause epilepsy. Serial voxel-based automated quantitative analyses are more sensitive than visual reading for detecting change in a lesion. Eloquent cortex can be identified with functional MRI (fMRI), with cautions about the precise location and extent of critical cortex. Tractography is useful for delineating critical white matter tracks as are MR venography and computerized tomography (CT) angiography for displaying veins and arteries. These data may be combined into a three-dimensional (3D) multimodal MR data presentation and displayed interoperatively to increase the precision and minimize the risk of neurosurgical treatment, and for the illustrations.

It is well-recognized that brain tumors may cause epilepsy; this may occur with both primary and secondary tumors, and those that are extrinsic to the brain as well as intrinsic. The primary issues that determine the optimal treatment plan for individuals with brain tumors that cause epilepsy are whether epilepsy is well-controlled with medication, or is refractory to treatment, and whether the priority for treatment is the tumor or the epilepsy. The surgical strategy will be different according to whether the priority is treatment of the epilepsy or of the tumor, and the extent of resection will be determined by proximity to eloquent cortex and critical white matter tracts (Gil-Robles & Duffau, 2010). Treatment that is directed primarily at treating the epilepsy depends on locating the epileptogenic zone and may require invasive electroencephalography (EEG) studies, as this area may not be the same, for example, as areas that show contrast enhancement, which are likely to be the priority for resection for primarily oncologic purposes.

Low grade gliomas (Fig.1) frequently present with an epileptic seizure (Ruiz & Lesser, 2009; Smits & Duffau, 2011; Thom et al., 2012). More than 50% of primary brain tumors are grade IV malignant glioblastomas multiforme (Fig.2), which spread rapidly and diffusely through the brain (Englot et al., 2012). A low grade primary brain tumor will have mass effect, low signal on T_1_-weighted images, and increased signal on T_2_-weighted and fluid-attenuated inversion recovery (FLAIR) sequences (Price, 2010). Cerebral metastases are overall many times more common than primary brain tumors (Fig.3). High grade primary brain tumors may also show surrounding edema, contrast enhancement, and an area of necrosis (Clarke & Chang, 2012). Cerebral metastases are frequently multiple, commonly occur at the gray–white matter junction, in watershed areas, show contrast enhancement with surrounding edema, and rarely cross the midline (Lee et al., 2012). Although cerebral metastases may present with an epileptic seizure, it is uncommon for troublesome refractory epilepsy to be a dominant feature for an individual with cerebral metastases.

**Figure 1:**
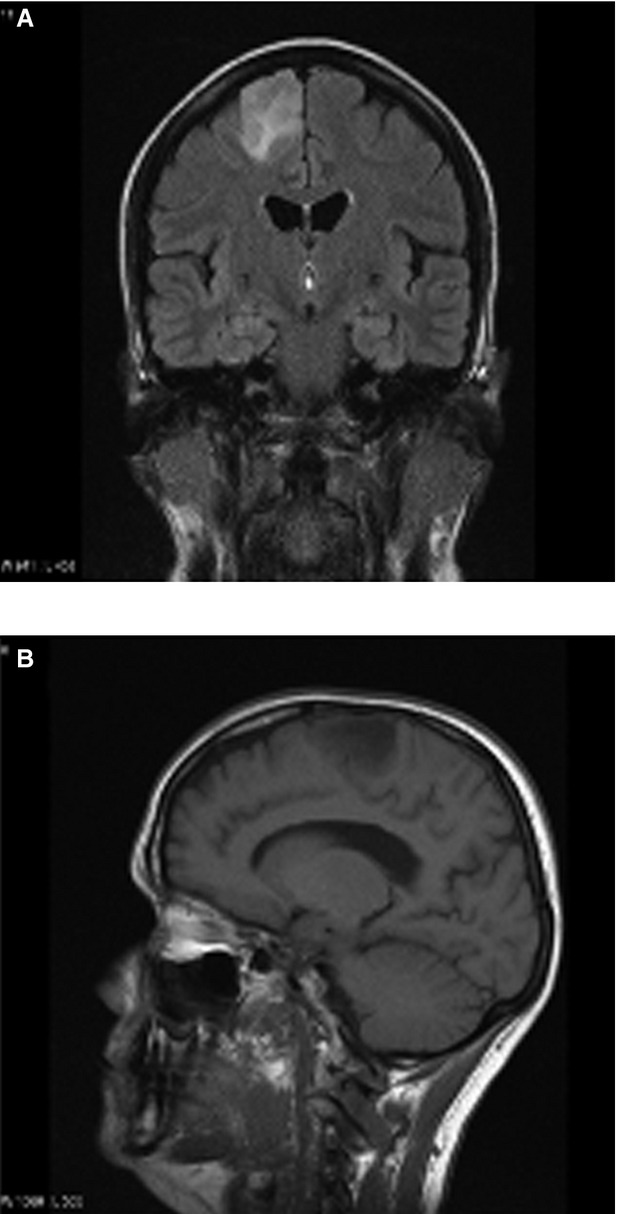
Coronal FLAIR (A) and sagittal T_1_-weighted (B) MR images of a low grade glioma in right superior frontal gyrus, causing refractory focal epilepsy.

**Figure 2:**
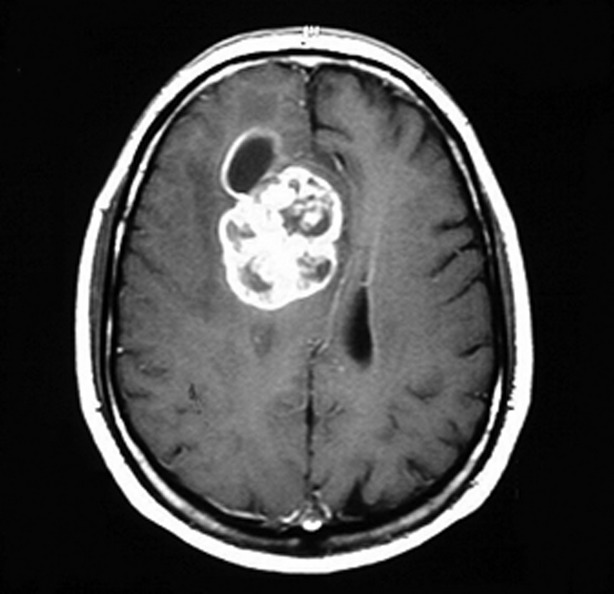
Glioblastoma multiforme, showing contrast enhancement.

**Figure 3:**
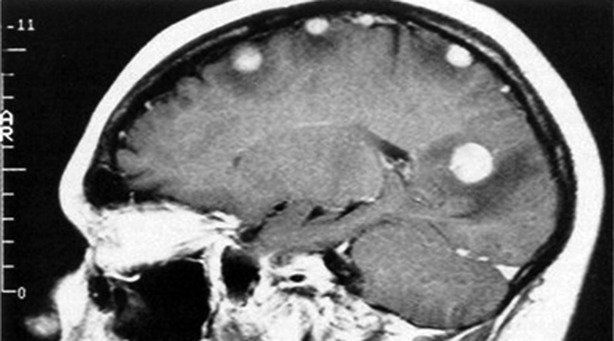
Multiple cerebral metastases, with contrast enhancement and surrounding edema.

A benign lesion, such as a dysembryoplastic neuroepithelial tumor (Fig.4) or ganglioglioma (Campos et al., 2009) may be structurally static, and if the epilepsy is well controlled with medication, a conservative policy is reasonable, particularly if removal of the lesion would carry a significant risk of causing neurologic deficit. In such a situation, follow-up magnetic resonance imaging (MRI) is recommended, ideally using the same protocol on the same instrument so that comparison is facilitated. Administration of intravenous contrast is helpful to help determine if there is evidence of malignant transformation. The interval between scans varies according to clinical circumstances. An initial interval of 6 months is reasonable, followed by an increase to intervals of 12 to 24 months, if the appearances are stable. It is important that the comparisons of sequential MRI scans are not just between the last pair, but also between the first scan taken and the last scan taken so that they are compared over an increasing interval, thereby maximizing the possibility of discerning subtle increases in size. Visual interpretation of scans is relatively insensitive and will not reliably pick up changes in volume of <15–20%. Accordingly, quantitative analysis with coregistration of scans is significantly more sensitive and is very appropriate if it has been decided that intervention will be made if there was an increase in lesion size (Liu et al., 2003; Burdett et al., 2006; Pallud et al., 2012).

**Figure 4:**
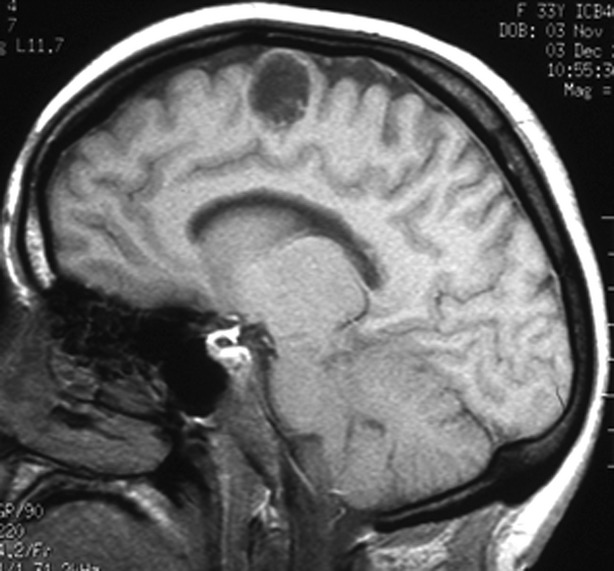
Dysembryoplastic neuroepithelial tumor, stable over many years. Epilepsy controlled with antiepileptic drugs.

Key in making a decision about the safety of the resection is the proximity of a lesion to eloquent cortex, particularly that subserving language, motor function, and somatosensory function and vision, and proximity to critical white matter tracts, particularly corticospinal tract and optic radiation. Although functional MRI (fMRI) with verbal fluency and verb generation paradigms is generally reliable for language lateralization (Binder, 2011; Williams et al., 2012; Janecek et al., 2013), these data are not currently reliable for localizing language cortex to determine the safe limits of surgical resection in the vicinity. It cannot be assumed that areas that do not show activation can be safely resected, and conversely, it cannot be assumed that areas that do activate are essential to maintenance of language function (Seghier et al., 2011). Additional language paradigms are being developed with the expectation of being able to better localize critical language areas in frontal and temporal lobes (Price, 2012).

An important caveat for the use of all fMRI studies in surgical planning is that the extent of eloquent cortex cannot be safely determined with fMRI, as the extent of activation evident on an image will be clinically affected by the threshold used for the image display.

If a low grade lesion is found, for example, in the anterior part of the language nondominant frontal lobe, resection may be recommended without further imaging or invasive electroencephalography (EEG) investigations. If, however, a lesion is close to primary motor cortex, identification of this with motor fMRI and tractography to define a corticospinal tract is helpful. If there is proximity, further anatomic definition of the area to be resected, and the areas that must be spared is helpful, using either an awake resection or stimulation mapping of eloquent cortex. If surgery is being carried out primarily because of refractory epilepsy and depth EEG recording is required to determine the epileptogenic zone, and the relation of this to eloquent cortex, the display of arteries and veins in a 3D multimodal imaging setup is invaluable to minimize the risk of causing a new deficit and of puncturing vessels with depth electrodes, thereby causing a hematoma.

Critical tracts, such as the corticospinal tract and optic radiation, are best defined preoperatively using probabilistic tractography (Fig.5; Guye et al., 2003; Yogarajah et al., 2009; Winston et al., 2012). Deterministic tractography is quicker, but does not give sufficiently reliable results, particularly for angulated tracks. The tractography data can then be presented in the eyepiece of the operating microscope, facilitating avoidance of critical structures by the surgery (Daga et al., 2012).

**Figure 5:**
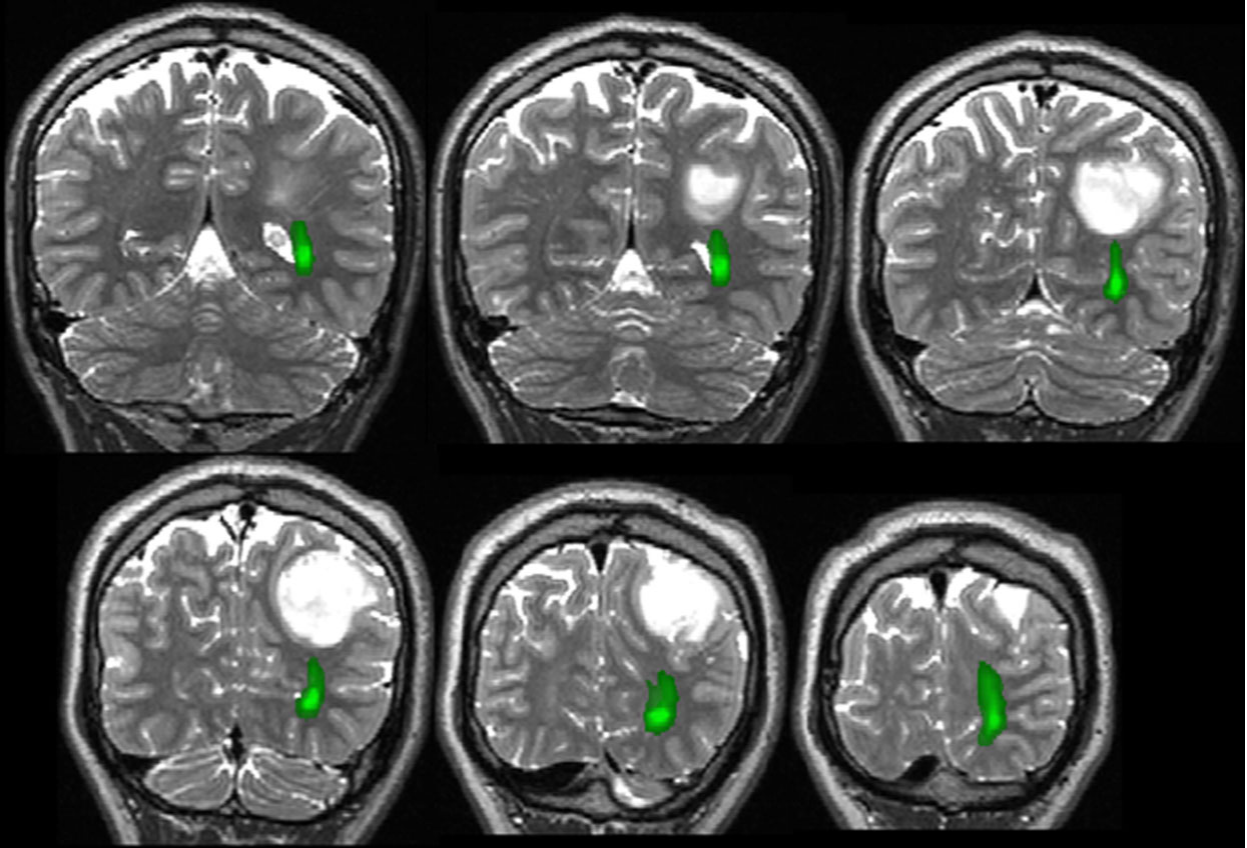
Optic radiation visualized with tractography (green), inferior to low grade tumor and lateral to lateral ventricle.

The use of interventional MRI, with the obtaining of MR sequences immediately prior to resection and before the craniotomy is closed, is helpful for ensuring complete lesion resection at the time of the surgery with the further advantage that intraoperative imaging allows correction for the brain shift that inevitably occurs after craniotomy, loss of cerebrospinal fluid (CSF), and removal of part of a mass lesion (Kubben et al., 2011). Intraoperative MRI sequences would typically include T_1_, T_2_, and FLAIR volume acquisitions for navigation, a high resolution T_2_ image, and diffusion-weighted imaging. The latter is most useful for coregistration with a preoperative fractional anisotropy map, which carries with it the representation of a tractographic representation of, for example, the optic radiation, which is then warped into an orientation that is corrected for the brain shift that occurs through the surgery (Daga et al., 2011).

## Disclosures

Prof Duncan has received Institutional support from UCB Pharma, Eisai, GSK, Janssen-Cilag, Cyberonics, MedTronic, and GE Healthcare. The authors confirm that we have read the Journal's position on issues involved in ethical publication and affirm that this report is consistent with those guidelines.
